# Comparative Overview of the Mechanisms of Action of Hormones and Endocrine Disruptor Compounds

**DOI:** 10.3390/toxics7010005

**Published:** 2019-01-24

**Authors:** Yves Combarnous, Thi Mong Diep Nguyen

**Affiliations:** 1 CNRS, INRA, Physiologie de la Reproduction & des Comportements, 37380 Nouzilly, France; nguyenthimongdiep@qnu.edu.vn; 2 Faculty of Biology-Agricultural Engineering, Quy Nhon University, Binh Dinh 820000, Vietnam

**Keywords:** endocrine disruptor, hormone, receptor, mechanism, risk assessment

## Abstract

Endocrine Disruptor Compounds (EDCs) are synthetic or natural molecules in the environment that promote adverse modifications of endogenous hormone regulation in humans and/or in wildlife animals. In the present paper, we review the potential mechanisms of EDCs and point out the similarities and differences between EDCs and hormones. There was only one mechanism, out of nine identified, in which EDCs acted like hormones (i.e., binding and stimulated hormone receptor activity). In the other eight identified mechanisms of action, EDCs exerted their effects either by affecting endogenous hormone concentration, or its availability, or by modifying hormone receptor turn over. This overview is intended to classify the various EDC mechanisms of action in order to better appreciate when in vitro tests would be valid to assess their risks towards humans and wildlife.

## 1. Introduction

Endocrine disruptor compounds (EDCs) are mostly synthetic molecules from industrial origin [1,2,3] but are also some natural molecules [4,5] that are present in the environment and promote adverse modifications of endocrine homeostasis in humans and/or in wildlife animals. EDCs raise serious concerns about their potential health impact.

Most of the receptors that are targeted by EDCs are nuclear receptors. These receptors are hormone-dependent transcription factors and, consequently, they exert long-term control of their target cells’ phenotype. Membrane receptor signaling can also be affected by EDCs but this potentially leads to a short-term effect, since their signaling pathways exert more acute effects in target cells. An interesting case is that of estradiol, that binds to a transmembrane receptor named GPER (or GPR30) in addition to its nuclear receptors ERα and ERβ [6]. Interestingly, the EDC bisphenol-A exhibits a higher affinity towards GPER than toward its nuclear ER receptors [7].

The understanding of EDCs’ mechanisms of action, as well as the extent to which their effects are responsible for health disorders, are the subject of scientific and public controversy. We present here information concerning EDCs compared to hormones in order to evaluate their particular properties and to estimate their potential risks for human and animal health.

In a previous article [8], we summarized the main mechanisms of action of EDCs. We present here a more precise view of the different mechanisms that EDCs can exhibit, including:

(1) Binding to a hormone receptor leading to activation of its signaling pathway;
(2) Binding to a hormone receptor leading to inhibition of its signaling pathway;
(3) Interactions with components of hormone signaling pathway downstream of a receptor;
(4) Stimulation or
(5) Inhibition of an endogenous hormone biosynthesis;
(6) Binding to circulating hormone-binding protein;
(7) Stimulation or inhibition of hormone-binding protein synthesis or degradation;
(8) Stimulation or
(9) Inhibition of hormone receptor expression.

**Figure 1:**
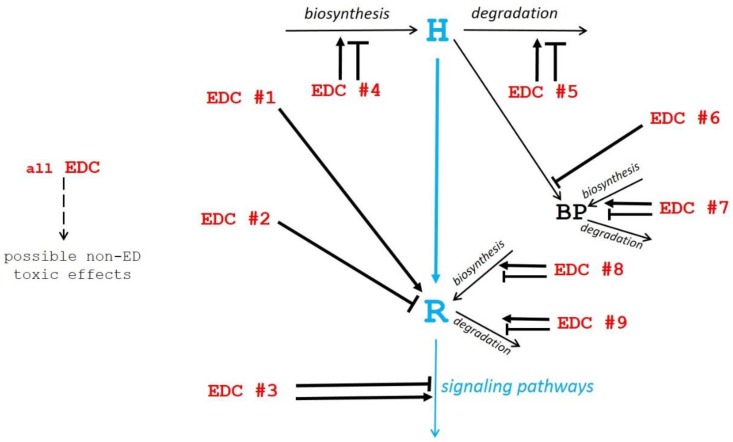
Schematic view of potential mechanisms of action of endocrine disruptor compounds (EDCs). The physiological hormonal mechanism is shown in blue. The diverse EDC mechanisms of action (EDC #1 to EDC #9 in red) as described in the text, are shown by black arrows pointing to their site of action (→ stimulation; ─┤ inhibition).

Among these mechanisms, only the first one is common within the mechanisms of any action relating to hormones. The other mechanisms (numbers 2 to 9) lead to imbalances in endocrine homeostasis that are not consecutive to a direct hormonal-type mechanism of action by EDCs. We have thus taken these various mechanisms into consideration and summarized them in Figure 1, to differentiate the different types of EDCs according to their similarities or differences compared to hormones.

## 2. EDCs Directly Exhibiting Hormonal Activity Through Receptor Binding (Mechanism 1)

The most obvious EDCs are those exhibiting a hormone-type mechanism of action, i.e., those able to bind to and activate a hormone receptor (mechanism number 1 above). How is that possible, since hormones are considered to exhibit high affinity and high specificity towards their cognate receptor? Because EDCs exhibiting structures that are different from those of hormones, can sneak into their binding site and interfere with their mechanism of action.

The present hormone receptor (HR) couples, in all today’s living species, are the fruit of evolution. Natural selection during evolution does not work on each HR couple individually (and neither on interactions involving each enzyme, structural protein or others) but on organisms exhibiting all possible sets of protein forms and thus, various capabilities to thrive in various environmental situations. Concerning HR couples, it is not sufficient that they exhibit high affinity but also that they do not interfere with others. It must thus have a high specificity so that hormones with almost similar structures (androgens vs. estrogens for example) do not interfere. Thus, evolution has not only selected individual efficient HR couples but also couples without mutual interferences, i.e., receptors that recognize their cognate ligand but also inhibit the binding of other molecules with very near structures. Only functional isolation of HR couples allows harmonious endocrine controls.

The massive introduction, in terms of number and quantity, of synthetic molecules having more or less the shape and size of hormones explains that some of them can lure the receptors and bind to them [9]. Indeed, receptors have evolved to recognize their cognate ligand but also to impede the binding of other endogenous molecules with near structures. The receptors are thus protected against interaction with endogenous molecules resembling hormones. However, they cannot be protected against interaction with brand new molecules never encountered before during evolution. In addition, these new synthetic molecules can be present in rather high quantities, and therefore can compete with genuine hormones in spite of their lower affinity towards receptors, compared to hormones.

Examples of this mechanism concern essentially xenobiotics interacting with hormone nuclear receptors. Indeed, these receptors have hormone-binding sites of rather small size that can potentially accommodate many synthetic organic molecules of industrial origin, but also natural molecules. Moreover, as these receptors exhibit transcriptional activity, their activation by xenobiotics can profoundly affect target cells phenotype. Some EDCs nevertheless interact with membrane receptors [10].

There are many examples of this straightforward classical mechanism, in which EDCs act like hormones by direct interaction and activation hormone receptors. Nevertheless, EDCs might be less efficiently degraded than the natural hormones and thus be more active in vivo because of a longer half-life in blood or in cells.

## 3. EDCs Directly Inhibiting Endogenous Hormone Action Through Receptor Occupation (Mechanism 2)

Among the new molecules resembling hormones and able to bind to receptors, some can freeze the receptors conformation in their inactive state, and thus antagonize endogenous hormone action (mechanism number 2 above). Through such a mechanism, exogenous molecules can clearly exert endocrine disruption. For example, polychlorinated biphenyls (PCBs) can suppress transcription through inhibiting the binding of T3 to the thyroid hormone receptor (TR) and consequently, by dissociating the transcriptionally active TR/retinoid X receptor heterodimer complex from the thyroid response element (TRE) [11]. Additionally, anti-estrogenic, anti-androgenic, anti-progesteronic, and anti-ER activities were detected in samples from wastewater treatment plants [12].

In this mechanism, EDCs are able to bind to receptors like hormones by exerting an antagonistic effect, in contrast to hormones. This mechanism of action can be tested in vitro but the cytotoxic effects of unknown substances can confound in vitro assays. This can make the interpretation of results difficult and uncertain, particularly when assessing antagonistic activity [13].

## 4. EDCs Interacting with Hormone Signaling Pathways (Mechanism 3)

A number of EDCs interplay with endocrine regulations through direct interaction. This means interaction is not with hormone receptors, but with hormone signaling pathway components downstream of receptor activation. Such molecules can exhibit structures that are largely different from those of hormones.

For example, fluoxetine (FLX) that is the SSRI (selective serotonin reuptake inhibitor) active substance in the Prozac™ antidepressant has been shown to modify a number of intracellular signaling pathways in various cell types [14,15,16]. Several bisphenols have been shown to interact with Ras small G proteins (particularly K-Ras4B) leading to the activation of the Ras signaling cascade, as shown by raised pERK and pAKT levels [17].

Atrazine, one of the most commonly used herbicides worldwide, acts as an endocrine disruptor by inhibiting cAMP-specific phosphodiesterase PDE4 [18] and thus, favors cAMP intracellular accumulation. Tolylfluanid impairs insulin signaling in human adipocytes through a reduction in insulin receptor substrate-1 (IRS-1) levels downstream from the insulin receptor [19].

The plasticizer di-(2-ethylhexyl)-phthalate (DEHP) is classified as an endocrine disruptor but also as an obesogen and has been shown to act through the peroxisome proliferator activated receptors (PPARs) [20], provoking downstream effects on AMPK, ERK1, ERK2 and ACC activation through phosphorylation. DEHP can also exhibit non-endocrine reprotoxic effects by directly affecting these pathways in gametes [21].

Recently, neonicotinoid pesticides have been shown to induce a change in *CYP19 (aromatase)* promoter usage in Hs578t breast cancer cells, leading to increased aromatase catalytic activity and the activation of MAPK 1/3 and/or PLC pathways [22]. This promoter usage change is similar to that observed in patients with hormone-dependent breast cancer. Another example concerns the effect of triclosan, a broad-spectrum antibacterial and antifungal compound, on ovarian [23] and testicular steroidogenesis through miRNAs which are involved in endocrine regulation and disease development in humans [24]. For example, the miR-6321/Map3k1-regulated JNK/c-Jun/Nur77 cascade contributes to a triclosan endocrine disrupting effect [25].

Histone methylation events are a general component of nuclear receptor mediated transcriptional regulation, for example in the testis [26]. DNA methylation of a Wnt2 promoter, under bisphenol-A (BPA) exposure, is implicated in preeclampsia-like effects in mice [27]. BPA also affects cell proliferation of human placental first trimester trophoblasts [28] and is thus of concern for the sensitive window that is fetal development.

In this mechanism, EDCs do not interfere with hormone receptors but downstream of them, at numerous possible sites which can be difficult to identify. Potentially, this type of mechanism should be detectable and quantitated in vitro in cell culture systems. It must be kept in mind that this mechanism can lead to direct, non-endocrine, and toxic effects (Figure 1).

## 5. EDCs Affecting Endogenous Hormone Concentration (Mechanisms 4 and 5)

Many molecules can exert endocrine disruption, not by interfering directly with hormone receptors, but by affecting, positively or negatively, endogenous hormone(s) biosynthesis (mechanism 4) or degradation (mechanism 5). Such molecules generally exhibit structures that are different from those of hormones, since they do not compete with hormones at the receptor level.

### 5.1. Mechanism 4

One example of this mechanism is that of BPA which, at a low dose, inhibits adiponectin secretion in vitro in human adipocytes [29,30,31,32]. It has been shown that EDC 4-nonyphenol (4-NP) inhibits the secretion of testosterone by Leydig cells stimulated by human chorionic gonadotropin [33] and triclosan induces Vascular Endothelial Growth Factor (VEGF) secretion by human prostate cancer cells [34].

### 5.2. Mechanism 5

Flame retardants such as polybrominated diphenyl ethers (PBDEs) have been described to act through the induction of hepatic enzymes involved in glucuronidation [11], thus potentially leading to an increase in T4 elimination and the lowering of its concentration in blood. Parabens, which are effective preservatives widely used in cosmetic products, inhibit 17β-hydroxysteroid dehydrogenase (17β-HSD) and consequently inhibit estrogen degradation [35], potentially leading to an increased hormone concentration in blood.

In this mechanism again, EDCs do not interfere with hormone receptors but, by affecting endogenous hormone concentration, impact either their biosynthesis or degradation. Such a mechanism has to be studied in vivo but can be tested in vitro when a specific step has been identified.

## 6. EDCs Affecting Endogenous Free Active Hormone Concentration (Mechanisms 6 and 7)

Many hormones, particularly the hydrophobic ones (steroids and thyroid hormones), are transported by binding proteins in blood. Since EDCs are generally hydrophobic, they are susceptible to compete with small hydrophobic hormones in relation to these transport proteins.

### 6.1. Mechanism 6

A number of EDCs directly interfere with hormone-binding transport proteins, thus competing with the endogenous hormones’ concentration in blood. For example, numerous chemicals have been shown to interact with SHBG (steroid hormone-binding protein) or AFP (α-fetoprotein) [36,37] and thus, able to interfere with steroid hormones transport and concentration in blood. The EDCs exerting their effect through this mechanism exhibit some structural resemblance with the hormones, so that they can compete with them for binding with hormone-binding transport proteins.

In this mechanism, EDCs do not compete with hormones at the receptor level, but at the level of their circulating binding proteins. They can thus exhibit structural resemblance with the hormones they compete with, and this competition can be studied in vitro.

### 6.2. Mechanism 7

Other EDCs affect the biosynthesis or degradation of hormone-binding transport proteins, so that both the total hormone concentration and/or its free active fraction can be affected. The EDCs acting this way can exhibit chemical structures very different from those of hormones. For example, PBDEs act through the down-regulation of the transport protein transthyretin (TTR) [11] and therefore can lower T4 concentration in blood.

Through this mechanism, many toxicants can be catalogued as EDCs because hormone-binding transport proteins are often synthesized and/or degraded by the liver, which, as a (degrading) organ, is the main target of toxicants.

## 7. EDCs Affecting Endogenous Hormone Receptor Turn-Over (Mechanisms 8 and 9)

### 7.1. Mechanism 8

Stimulation of endogenous hormone receptors is a way by which a number of EDCs interfere with endocrine homeostasis. BPA has been shown to stimulate leptin receptor expression in ovarian cancer cells in vitro [31]. Cadmium exposure of endothelial HUVEC cells in vitro induced a significant increase of estradiol receptor β (ERβ) and Cyp19a1 enzymes at both mRNA and protein levels, while a drastic dose-dependent decrease of androgen receptor (AR) expression levels was observed after 24 h of exposure [38].

### 7.2. Mechanism 9

Inhibition of receptor expression is also a mechanism responsible for EDC alteration of the endocrine system. It has also been described that a low oral dose of BPA given to rats can inhibit estrogen receptor expression in their hypothalamic cells [39]. Likewise, inhibition of androgen receptor expressions by BPA has been described in vivo [29] and in vitro in cells from breast or prostate cancer patients [40]. Such an inhibition has also been observed in newborn rats exposed to BPA and was attributed to hypermethylation of the androgen receptor promoter [41]. Moreover, BPA can selectively affect the expression of the ecdysone receptor gene expression in insects [42], whereas it promotes a decrease in ERα, ERβ and GPR30 in fetal mammary gland [43].

In these mechanisms, EDCs generally do not need to resemble hormones to exert their adverse effect by modifying receptor availability. Nevertheless, receptor synthesis and/or degradation are often controlled by its cognate hormone [43]. In this case, EDC structural similarity with hormones can be responsible for this effect. These mechanisms can potentially be identified and studied in vitro, using cell culture assays.

## 8. In Vitro Tests vs. In Vivo Tests

Mechanisms 1 and 2 are relatively easily amendable to in vitro tests to replace in vivo tests, making use of living animals [44,45,46]. Mechanisms 8 and 9 can also be potentially studied in vitro. In vivo tests in aquatic animals reproducing EDC concentrations recorded in polluted places in the environment are particularly useful [47,48], but not always easy to interpolate to terrestrial species, including humans.

The limited capabilities of in vitro models to metabolically activate or inactivate xenobiotics may lead to misinterpretation of the in vitro data if such information is missing [49]. These authors have shown that HC11 cells did not show any biotransformation capability, while the major biotransformation pathways in HepG2 and MCF7 cells were conjugated to sulfate and, to a lesser extent, glucuronic acid. These results suggest that HC11 cells should be a valuable cellular system to study the intrinsic estrogenic activity of the tested compound. In these cells, it is thus the concentrations of EDCs in active form that must be taken into consideration. Using HepG2 and MCF7 cells that are able to metabolize activity can help to take into account part of the metabolic fate of the tested compound that occur in vivo.

Since a number of metabolizing enzymes are poorly or not at all expressed in standard in vitro systems, their use in endocrine disruptor testing may result in false negatives for compounds in which bioactivation is a prerequisite.

In vitro and in vivo tests are complementary but in vivo tests have to be kept at a minimum for ethical reasons, providing, nevertheless, that in vitro tests give sufficient reliable information.

## 9. Endocrine Disruption vs. Other Toxicological Mechanisms

Molecules with recognized endocrine disruption activity can also have additional adverse effects through other toxicological mechanisms. They can directly be cytotoxic or reprotoxic (i.e., direct alteration of gametogenesis or other reproductive steps) [50], or teratogenic or genotoxic (alteration of DNA: either by epigenetic alterations or through mutations), possibly leading to cancers independent of endocrine-related cancers. For example, BPA exhibits cytotoxic and genotoxic effects not related to its EDC properties [50,51,52]. Likewise, dioxin that acts as an EDC through the aryl hydrocarbon receptor (AhR) [53] has also been shown to be a potent genotoxic [54]. Although these cytotoxic and genotoxic effects are generally observed at higher concentrations than endocrine-disturbing effects, this possibility must be taken into consideration.

As with the other toxicants, EDCs exhibit longer effects during early developmental steps, such as embryonic, fetal, neonatal, childhood, and puberty periods [52,55]. Obviously, defects during developmental steps will have consequences during the whole life of the exposed individual [56] and sometimes in its descendants, as these effects often occur through epigenetic mechanisms [57]. Whatever the mechanism in action, it would be wise to study EDC effects in these sensitive windows and observe the consequences in adults. Nevertheless, numerous and long-term experiments for testing individual EDCs or mixtures in animals would be nearly impossible for all suspected molecules. It is therefore advantageous to classify individual molecules according to their disturbing mechanisms, in order to get a better analysis of their synergies in mixtures.

## 10. EDCs Mechanisms of Action and Risk Assessment

As stated in the WHO-UNEP 2012 document [58], EDCs represent a challenge as their effects depend on both the level and timing of exposure, being especially critical when exposure occurs during development. “Risk assessment” is the term generally used to refer to the characterization of the potential adverse effects of exposure to hazards. The evaluation of EDC risk assessment is an issue leading to controversies [58,59,60,61].

Therefore, the timing of exposure and of its acceptable quantitative limits are of major interest to assess risk [62]. Nevertheless, the existence of dose-thresholds for endocrine disruptors continues to be debated [63,64,65,66,67] because non-monotonous dose response (NMDR) curves are often considered as an intrinsic property of EDCs in the non-scientific press and general public. It is rather a property derived from the complexity of endocrine regulations [64]. It remains nevertheless, that it can be difficult to distinguish a valid true threshold from an apparent threshold, which merely arises from the limits of detection of the experimental system used.

If a molecule exhibits a U-shape dose-response curve in a given experimental system, the U descending branch of the curve should be used as the basis for determining the threshold if the registered response is related to the adverse effect of the molecule. This can lead to exceedingly low limits but, at least, this is more satisfying than, by principle, refusing any limit. Of course, the determination of the control value in the total absence of the molecule under test is primordial to demonstrate a significative, positive or negative, effect at these very low doses.
